# Three-dimensional exploration of the chicken embryo, a comparative study of light sheet and histological visualisation

**DOI:** 10.1371/journal.pone.0320483

**Published:** 2025-04-01

**Authors:** M. W. Smallridge, T. E. Aktepe, M. J. C. Coppo, P. K. Vaz, A. Diaz-Méndez, C. M. Murray, G. Segal, J. M. Devlin, C. A. Hartley

**Affiliations:** 1 Faculty of Science, Asia-Pacific Centre for Animal Health, Melbourne Veterinary School, The University of Melbourne, Melbourne, Victoria, Australia,; 2 Facultad de Ciencias de la Vida, Escuela de Medicina Veterinaria, Universidad Andres Bello, Concepcion, Biobio, Chile; 3 Biological Optical Microscopy Platform, The University of Melbourne, Melbourne, Victoria, Australia; Goa University, India, INDIA

## Abstract

Ultramicroscopy has offered new avenues into the visualisation of tissues within animal models, providing three-dimensional visualisation through the use of light sheet fluorescence microscopy. This study aimed to develop and apply an optical tissue clearing method to investigate the application of light sheet fluorescence microscopy to image late-stage chicken embryos, and compare anatomical visualisation to traditional histological staining. Seventeen-day old specific pathogen free embryos were collected, fixed, and sectioned. Haematoxylin and eosin stained sections were prepared for histology, while light sheet imaging required the tissues to be optically clear. For this, an ethyl cinnamate-based method was utilised, allowing for acquisition of clear, unobstructed three-dimensional images of significant organ structures and systems using only autofluorescence. The use of established histological techniques provided anatomical mapping of structures between familiar histology images and the three-dimensional light sheet images. Rendering of organs using light sheet imaging provided contextual insights into the surrounding tissues and physiological architecture of major organ structures and systems. This was most apparent through the identification of the pulmonary vein and rendering of a volumetric projection of the vasculature branching within the lung and the subsequent merging of vasculature into the left side of the heart. Overall, the visualisation of the chicken embryo was enhanced by combining traditional histology with the information gained by three-dimensional light sheet fluorescence microscopy.

## Introduction

Animal models are often used in biological research to characterise disease pathophysiology [1–5], and to study anatomy and embryo development [6]. These models have trended towards mammalian species due to their pathophysiological relevance to humans and their subsequent translation to clinical trials [7]. Mice are the most common animal model used throughout biomedical research, attributed to their anatomical, physical, and genetic characteristics such as size, generation interval, and susceptibility to human infectious diseases [3,7].

*In ovo* models are used to study embryonic development [8, 9] and as a substrate to isolate and propagate bacteria and viruses, and understand tumour biology [10–12]. Primary isolation of a range of agents from clinical cases in human and veterinary medicine occurs *in ovo* [13–16], as does the production and propagation of vaccines for diseases of medical and veterinary importance [17, 18]. *In ovo* vaccination with live attenuated vaccines is also broadly applied in commercial hatcheries for its convenience of delivery and ability to provide early protection against important pathogens of poultry [19–21]. As such, understanding the available methods for visualisation of the chicken embryo will be useful to understand the structures and tissues that are important to characterising the tropism, mechanism and distribution of infectious agents within this system.

Visualisation of tissues enhances understanding of disease pathophysiology for anatomical investigations at both the micro- and macroscopic scale. Imaging on a macro scale provides the context of the target tissue *in situ*, using techniques such as positron emission tomography (PET), computed tomography (CT), and magnetic resonance imaging (MRI) that rely on radiation or tissue density for imaging [4,22]. Virological research typically utilises methods such as conventional histopathological staining of paraffin embedded sections [6,23] or confocal microscopy [5,24,25] for visualisation at the cellular level. To achieve this level of resolution, significant sectioning is needed, resulting in some level of mechanical trauma to the tissues and artefacts from handling [26]. Although these techniques can provide cellular resolution, sectioning removes the spatio-morphological context to the surrounding tissue and systems. Recent developments in ultramicroscopy, such as light sheet fluorescence microscopy (LSFM), have enabled three-dimensional (3D) imaging of larger tissues at cellular resolution, without the need for significant sectioning [5,27–33].

Light sheet fluorescence imaging illuminates cleared tissues with a thin sheet of light across a single plane. A two-dimensional (2D) image is then taken of the single illuminated plane. This process is repeated throughout the depth of the tissue, and the series of 2D images are stacked digitally to create a full 3D image of the tissue [34]. This allows for visualisation and analysis *in situ* to provide context to critical structures of interest. Although LSFM does have limitations with specimen size, primarily along the z-axis, whole organism imaging has been achieved with various tissue optical clearing (TOC) methods [30,33,35].

For a sample to be imaged using LSFM, the tissue needs to be optically clear to provide an unobstructed path for the light sheet. Tissue optical clearing methods vary greatly and selecting the correct technique depends on both the tissue type and specimen size [36]. Organic solvent methods such as DISCO and ethyl-cinnamate (ECi) are relatively rapid and can be applied to clear a range of tissue types, however the dehydration that is required results in tissue shrinkage. Alternatively, aqueous tissue clearing methods, such as CUBIC have longer clearing times and detergents are required to clear more lipid dense tissues. As aqueous methods do not require a dehydration step, there is no tissue shrinkage, making their application preferential when shrinkage needs to be minimised. Despite the longer clearing time, aqueous solutions preserve the signal of fluorescence protein better that organic solvents and minimise tissue distortion [36–38]. Full-body clearing of mice has been achieved with both organic solvent and aqueous techniques [30,33,35].

The aim of this study is to investigate the application of LSFM in conjunction with traditional histopathology by developing a simple and inexpensive tissue optical clearing protocol to visualise late-stage chicken embryos.

## Materials and Methods

### Ethics

All animal experiments conducted in this study were approved by the University of Melbourne Animal Ethics Committee under Ethics Application number 24712.

### Incubation

Four specific pathogen free (SPF) white Leghorn chicken eggs (Australian SPF Services) were incubated at 37°C, 45% humidity and turned once an hour. Eggs were initially candled at 10 days of incubation (doi) and then daily from 12 doi onwards to assess viability. Euthanasia was performed at 17 doi by placing eggs at 4°C overnight.

### Embryo preparation

**Fig. 1:**
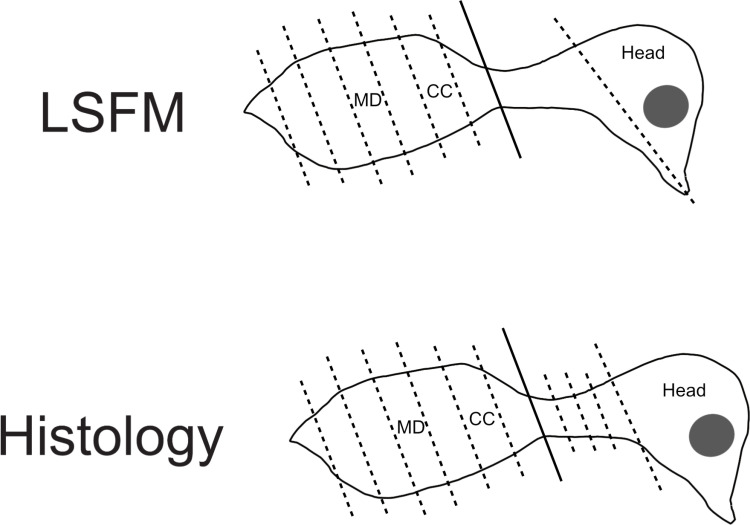
Line schematic of embryo sectioning for both LSFM (top) and histological (bottom) visualisation techniques. Solid line depicts initial cut for embryo preparation. Dotted lines demonstrate post-fixation sectioning. The sections visualised for this study are labelled. CC =  cranial coelom, MD =  mid coelom. Median sectioning of the head not shown.

The embryo was removed from the shell and placed in a petri dish, with the legs and wings then removed with a scalpel and discarded. The head was removed with a cut at the base of the neck, near the coelomic inlet (Fig. 1). The torso and head sections were then placed in 4% (w/v) solution of paraformaldehyde (PFA) in distilled water at 4 °C for two weeks.

## Sample preparation

### Histology

**Fig. 2:**
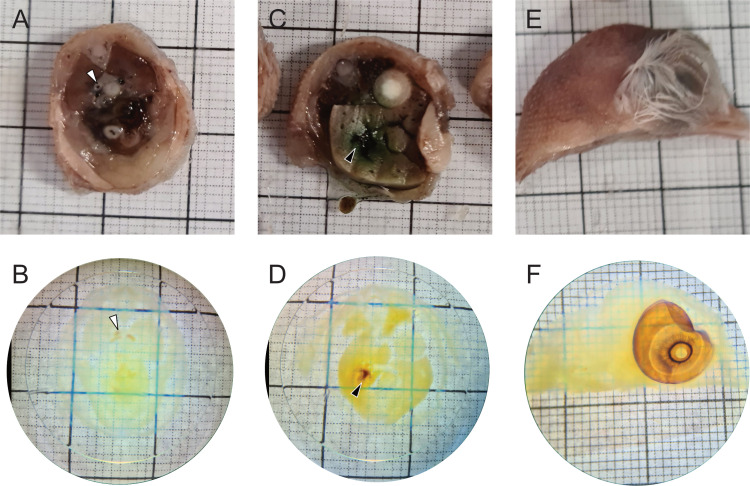
Representative transverse sections from day 17 chicken embryos. (A-**B)** Cranial coelom, (C-D) mid-coelom, (E-F) and head – lateral aspect of the right hemisection (A, C, E) prior and (B, D, F) post tissue optical clearing. One mm grid shown behind sections with 10 mm markings in bold. White arrowhead – descending aorta, black arrowhead – gall bladder.

Embryos were sectioned prior to histological preparation. Feathers were removed by gently wiping the embryo with a paper towel or by plucking. Using a scalpel, the torso was cut into a series of transverse sections approximately 5 mm thick, with a 1 mm grid used to guide section thickness (Fig. 2). The head was removed from the neck at the base of the mandible before being sectioned in the median plane. All sections were placed in cassettes and left in 70% w/v ethanol overnight to be processed by the Melbourne Histopathology Platform.

### LSFM preparation and tissue clearing

Sectioning of the torso was identical to that of the histology embryos. Processing of the head involved the removal of the neck and mandible from the cranium using a rostro-caudally directed cut *via* the oral cavity with a scalpel (Fig. 1). The cranium was then sectioned through the median plane to produce two even halves.

Initially, sections were immersed in 10% v/v hydrogen peroxide diluted in phosphate buffered solution (PBS: 0.137 M NaCl, 2.7 mM KCl, 8.1 mM Na_2_HPO_4_, 1.4 mM KH_2_PO_4_; pH 9.0) and placed in a water bath for 3 hours at 55 °C with occasional mixing, then immediately rinsed twice with PBS (pH 9.0) and incubated on a rotating platform (100 rpm) in fresh PBS (pH 9.0) for 30 minutes at room temperature. Dehydration was achieved through a series of five 24-hour incubations in ascending isopropyl alcohol (IPA, Chem Supply) concentration solutions of 30%, 50%, 70% v/v in MilliQ water respectively. The final 2 dehydration steps were both in 100% v/v IPA. Dehydration was performed on a rotating platform (100 rpm) at 4 °C.

Once dehydrated, embryo sections were removed from the IPA and immersed in undiluted ECi (Sigma Aldrich, Cat No. 112372) in glass jars. Sections were incubated on a rotating platform (100 rpm) at room temperature until completely cleared (12-24 hours).

### Image acquisition

Light sheet fluorescence images were taken using a LaVision Ultramicroscope II fitted with an Olympus MVX-10 Zoom body, an Olympus MVPLAPO 2x objective, and a dipping cap with a working distance of 10mm. Images were acquired with an Andor Neo sCMOS camera. Light sheet width of 60% and a sheet numerical aperture value of 0.052. Software used for acquisition was ImSpector Pro version: 5.1.363 (Lavision Biotec GmbH, Bielefild). The laser intensity was set at 30% and 40% for the 561 nm and 640 nm, respectively. Images were acquired sequentially with a z-step of 10 µm.

Imaging was initially performed at wavelengths of 405, 488, 561 and 640 nm. Autofluorescence of tissues at wavelengths of 405 and 488 nm did not provide enough signal above background levels to warrant further acquisition at these wavelengths. Autofluorescence was similar with both 561 and 640 nm lasers so image acquisition was performed at both wavelengths, with resolution and imaging depth observed in the 3D projections used as the final determinant of preferred autofluorescence wavelength.

### Image processing and analysis

Image processing and 3D rendering of LSFM images was conducted on a Hewlett Packard Z8 Xeon workstation. The system was powered by two Intel Xeon 6234 3.3 processors, contained 256 GB of RAM and a NVIDIA RTX A6000 graphics card. Stitching of images was performed using Imaris Stitcher and Imaris v10.0 software (Oxford Instruments, RRID:SCR_007370) was used for 3D and 2D image analysis of LSFM images.

Image processing was composed of three main steps: background subtraction, manual tracing, and surfacing. The background subtraction tool applied a Gaussian filter to define the background at each voxel and then performed a baseline subtraction of this variable background (Imaris reference manual 10.2, http://www.bitplane.com/download/manuals/ReferenceManual10_2_0.pdf). Heart, lungs, kidneys, and liver were manually traced every 50 µm along the XY axis from a 10 µm thick maximum intensity projection (MIP). The bifurcation of the pulmonary vein from the lungs to the heart was traced along the XZ axis every 50 µm using a 9.59 µm MIP. Traced selections had their fluorescence isolated to a new channel before surfacing the organ of interest using autofluorescence within the isolated channel. Vasculature branching within the left lung was surfaced without manual tracing, using only autofluorescence from the 561 nm channel. Imaris images available at https://doi.org/10.26188/28038932 [39].

## Results

### Tissue optical clearing

The ECi based TOC methodology utilised in this study was able to clear most tissues of the 17 doi chicken embryo sections. All sections and tissues appeared near-transparent after bleaching in hydrogen peroxide, dehydration, and incubation in ECi (Fig. 2). Tissues of interest (TOI) for this study were large, easily identifiable organs and their subsidiary vasculature to demonstrate LSFM imaging across several tissue types and organ systems. These included the heart and lungs in the cranial coelom (Fig. 2A and B), the liver and kidneys in the mid coelom (Fig. 2C and D), and the eye and nasal conchae in the head (Fig. 2E and F). Pigmentation remained within the gall bladder (Fig. 2D) despite bleaching.

### Cranial coelom

**Fig. 3:**
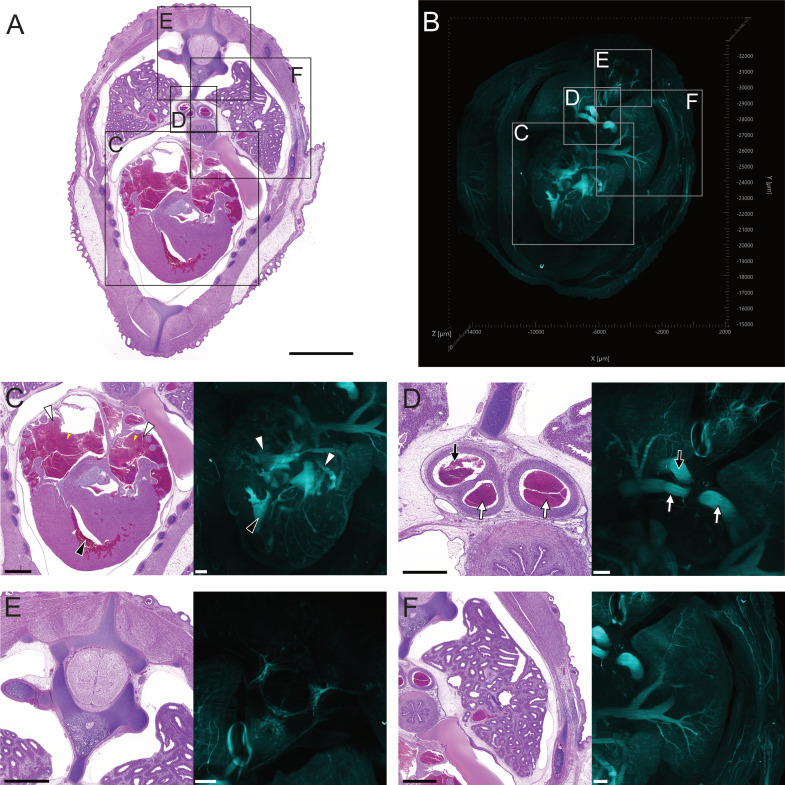
Comparative visualisation of major anatomical structures in the cranial coelom of day 17 chicken embryos. All images are viewed from cranial perspective. (A) H&E-stained histology section taken from a transverse cranial coelomic section of a day 17 chicken embryo, scale bar 3000 µm. (B) Orthogonal 3D LSFM fluorescence image of entire transverse cranial coelomic section from a day 17 embryo. All LSFM images acquired using autofluorescence at 561 nm. Boxes indicate areas of interest for major anatomical structures (C) heart; atria (white arrowheads) and right ventricle (black arrowhead) (D) vasculature; pulmonary arteries (white arrows) and descending aorta (black arrow) (E) vertebral column (F) left lung. Scale bars for histological image D is 500 µm, while histology images in C, E, F are 1000 µm. Scale bars for LSFM images C-F are 400 µm. (https://doi.org/10.26188/28038932).

The histology of the cranial coelom of the embryo (Fig. 3A) visualised landmark structures such as the spine (Fig. 3E) to provide an anatomical map for the LSFM images. The heart, descending aorta and pulmonary arteries, vertebral column, and lungs (Fig. 3C–F) were clear and easily identifiable. Blood was consistently found throughout the vasculature and organs in all histology sections, with blood cells staining a dark purple (Fig. 3A, C and D).

The equivalent 3D image acquired by LSFM provides a similar global representation of TOI within the coelom (Fig. 3B). The use of histological visualisation as a map for the unfamiliar 3D fluorescence projection enables the orientation and identification of TOI (Fig. 3C-F). Vasculature in the embryo was consistently located through its fluorescence contrast to surrounding tissue when imaged with LSFM at 561 nm. The remaining blood within atria and ventricles of the heart had similar autofluorescent characteristics when imaged (Fig. 3C).

**Fig. 4:**
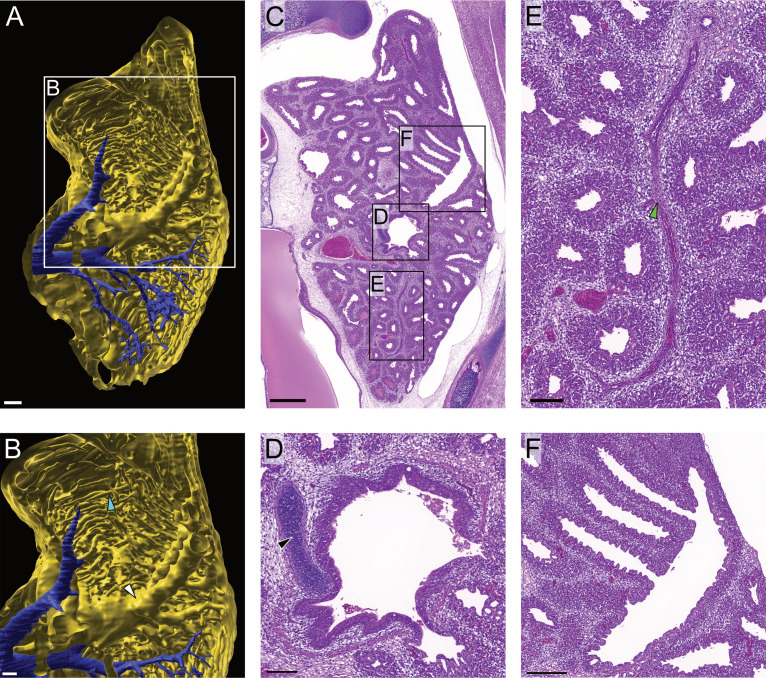
Representative LSFM and histological images of the left lung from day 17 embryos. All images are shown from the cranial perspective. (A) 3-dimensional representative model of a lung (yellow) and pulmonary vein branching (blue) from a day 17 chicken embryo. Volumetric projections modelled from autofluorescence at 561 nm wavelength (scale bar 300 µm). (B) Enlarged image of highlighted area in (A) showing thick bronchi (white arrowhead) and smaller bronchioles (blue arrowhead) within lung (scale bar 100 µm). (C) Transverse section of the lung in the torso of a day 17 chicken embryo stained with H&E (scale bar 500 µm). Boxed areas include (D) bronchi with hyaline cartilage (black arrowhead) scale bar 200 µm, (E) branching vasculature within the lung (arrowhead) (scale bar 100um), and (F) branching of the bronchioles (scale bar 100 µm). (https://doi.org/10.26188/28038932).

**Fig. 5:**
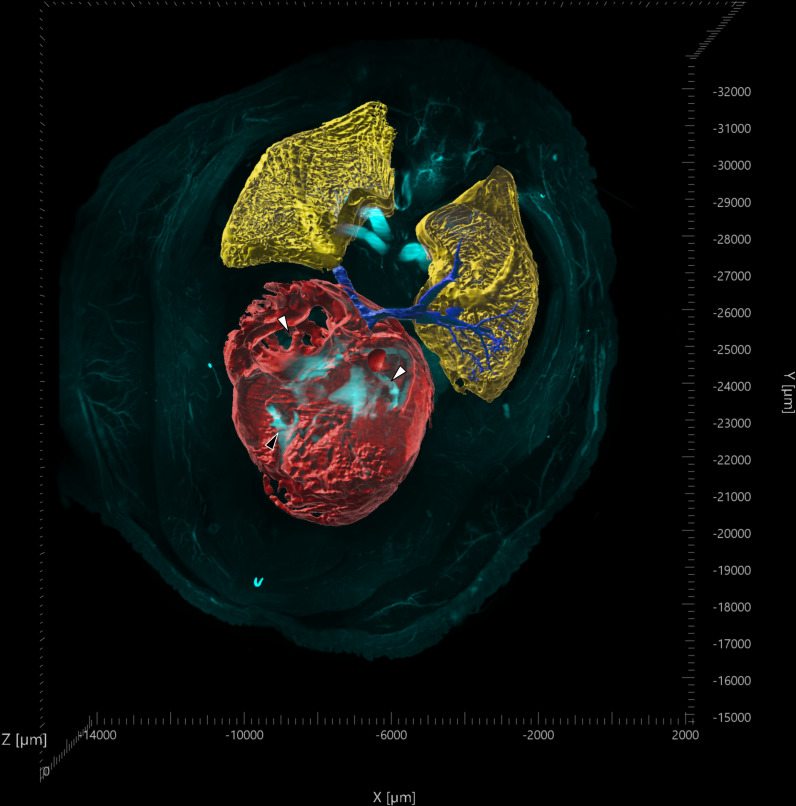
LSFM 3D fluorescence projection of transverse section of the cranial coelom from a chicken embryo. Autofluorescence at 561 nm (cyan) was used for volumetric projections of heart (red); atria (white arrowheads), right ventricle (black arrowhead), lungs (yellow), and pulmonary vein (dark blue). (https://doi.org/10.26188/28038932).

The H&E stained sections provided cellular resolution of anatomical structures such as the cartilage of the bronchi, with the epithelium in the lumen of the bronchioles clearly shown, although cilia were absent in the bronchi and bronchioles (Fig. 4C–F). Branching of vasculature, bronchi and bronchioles throughout the lung could be visualised as volumetric projections (Fig. 4A and B). Additional context was provided by both the 3D nature of the images and the ability to render TOI as volumetric projections (Fig. 5). Rendering of the lung, heart, and pulmonary vein into volumetric projections from the LSFM images was achieved using autofluorescence at 561 nm. This provided spatio-morphological context to the relationship between the vasculature and the respiratory system. Merging of the pulmonary vein from the lungs and into the heart could be rendered from its origin in both organs, with differentiation between veins and arteries possible due to vascular entry and location into the heart (S1 Video).

### Mid coelom

**Fig. 6:**
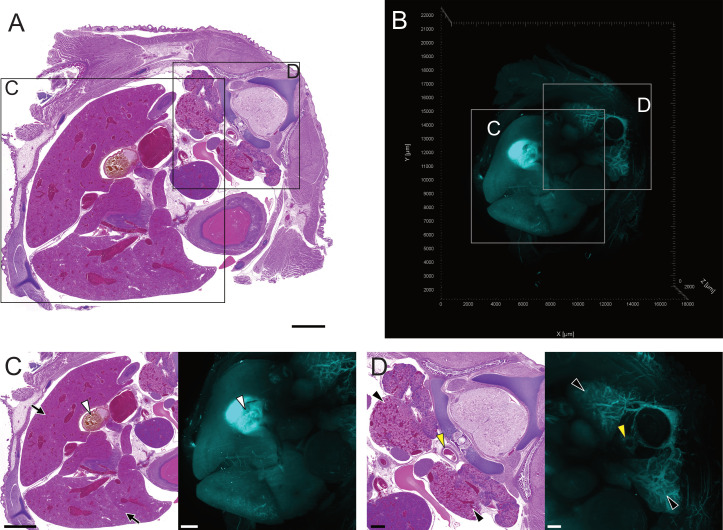
Representative images of a transverse section of the mid coelom from day 17 chicken embryos. Images were acquired using histological (A) and LSFM techniques (B). LSFM images display autofluorescence (cyan) at 561 nm wavelength from an orthogonal perspective. Areas of interest are labelled with boxes and magnified for direct comparison between imaging techniques (C-D). Areas of interest include: the liver (black arrows, C) with heavy pigmentation of the gall bladder visible through both techniques (white arrowhead), and the kidneys (black arrow heads) with vasculature around the spine visualised using LSFM and into the descending aorta (yellow arrowhead) (D). Histology scale bars: A =  2000 µm, C =  2000 µm, D =  500 µm; LSFM scale bars =  1000 µm. (https://doi.org/10.26188/28038932).

Histological staining displayed saturation of the liver and kidneys with blood cells, both within the organ and peripheral vasculature (Fig. 6A, C and D). The presence of blood throughout the embryo was consistent across both imaging methodologies, with vasculature clearly identifiable within the LSFM images due to the retention of blood in the kidneys (Fig. 6B and D). Heavy pigmentation of the gall bladder is seen in the histological images (Fig. 6C). This did not impact LSFM image acquisition for any of the surrounding tissues or structures within the section. Although increased fluorescence in the vasculature was consistent throughout all LSFM images, TOC was sufficient to enable even illumination across the samples. Remaining pigment within the gall bladder had minimal impact on the acquisition of clear images of the overall tissue.

**Fig. 7:**
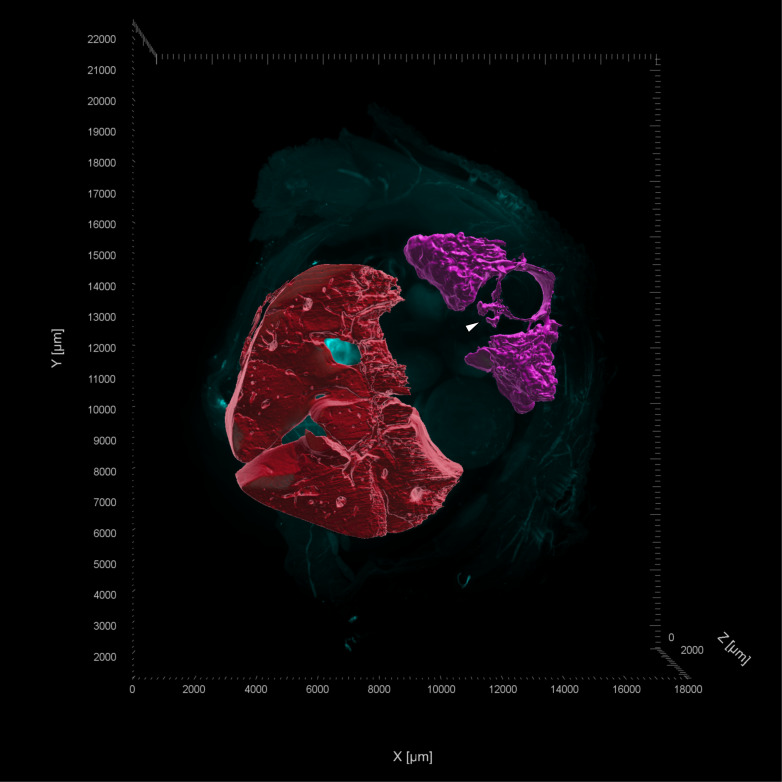
LSFM image of a transverse section from the mid coelom of a day 17 chicken embryo. LSFM images display autofluorescence at 561 nm (cyan) which was used for volumetric projections of the kidneys (pink), descending aorta (arrowhead) and liver (red). (https://doi.org/10.26188/28038932).

As in the cranial coelom, autofluorescence of the vasculature at 561 nm in the mid coelom section highlighted the blood supply in the kidney and descending aorta when viewing the 3D fluorescent images (Fig. 6D). This aided in modelling the kidneys, and blood supply originating from the dorsal aorta, as volumetric projections (Fig. 7 and S2 Video).

Clear images of the liver were defined in both LSFM and H&E histopathology (Fig. 6C). In both cases, the gall bladder remains heavily pigmented (Fig. 6C). The histology and LSFM images both prominently featured the distinctive vasculature pattern. Unlike the rest of the embryo sections, the vasculature of the liver did not fluoresce at a higher intensity than that of the surrounding tissue and other vasculature in the section (Fig. 6C). Regardless, the vasculature is a key identifiable characteristic when the organ was modelled as a volumetric projection (Fig. 7).

### Head

**Fig. 8:**
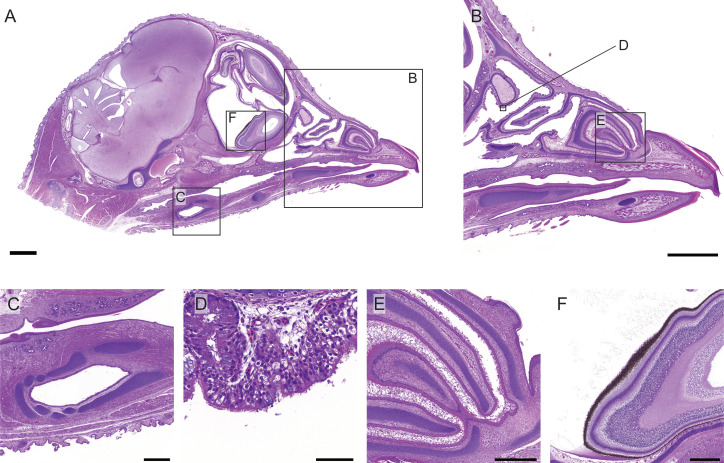
Histology images of sagittal section of a day 17 chicken embryo head stained with H&E. (A) Section of the median plane from the right hemisection of the head stained with H&E (scale bar 2000 µm). (B) Higher magnification image of the beak and mouth, areas of interest are depicted with boxes (scale bar 2000 µm). (C) Laryngeal and tracheal cartilage (scale bar 500 µm), (D) surface of caudal nasal concha (scale bar 50 µm), (E) rostral nasal concha (scale bar 500 µm), (F) pigmented ocular cells (scale bar 500 µm).

Histological staining of the head provided high resolution images of key anatomical structures. The sagittal sectioning of the head allowed for its use as a structural map along the median plane (Fig. 8A) using the eye as the major landmark. Visualisation of the conchae within the nasal passage (Fig. 8B), in addition to the trachea and laryngeal cartilage (Fig. 8C) was achieved. Microscopic visualisation of the epithelial layer within the nasal passage and trachea displayed an inconsistency in the location of development of cilia (Fig. 8D). The nasal plug is still apparent in the nasal conchae, with the canalisation process identifiable (Fig. 8E). Morphology of the brain and eye were visualised at the cellular level with histological staining, and the ocular pigment was clearly visible, maintaining its dark black colour (Fig. 8F).

**Fig. 9:**
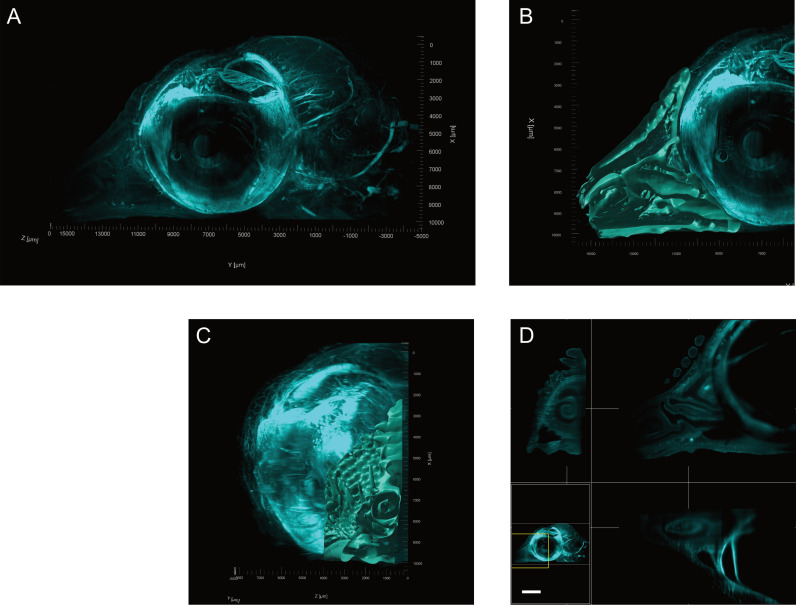
Representative LSFM fluorescence projections the chicken embryo head. (A) 3D fluorescence projection of right head hemisection from a day 17 chicken embryo imaged using LSFM techniques. All images acquired using autofluorescence at 561 nm. (B-C) Volumetric projection of the beak and nasal conchae viewed from the (B) medial and (C) ventral aspects. (D) 2D maximum intensity projection (9.69 µm) cross section of the beak. Axes displayed clockwise from top left are ZY, XY, XZ. White lines connecting sections display location of cross sections in adjacent images. (scale bar 1000 µm). (https://doi.org/10.26188/28038932).

Pigment in the eye was cleared sufficiently to acquire clear 3D images of the head using LSFM. The brain and eye were identified and compared with the histology. Consistent with previous LSFM images, the vasculature in the brain contrasted to that of the surrounding neural tissue (Fig. 9A). Light sheet imaging produced clear, 3D images of the nasal passage, with the turbinates visualised in 2D in each of the three axes (Fig. 9D), and as a 3D structure (Fig. 9B, C and S3 Video). The mapped intensity projections from each axis provided similar detail to the histology sections (Fig. 8), although the ability to view the complexity of the 3D structure in 2D from each of the XY, XZ and YZ planes allows for more conclusive identification of the turbinates (Fig. 9D).

## Discussion

This study aimed to investigate the application of a TOC methodology to LSFM techniques for the visualisation of late-stage chicken embryos, and to make comparisons with traditional histological tissue preparation. Light sheet imaging requires transparent tissue sections for successful imaging. Pigmentation from the heme in blood, and the heavy pigmentation within the eye can interfere with the ability to achieve the transparency required for LSFM [40], as such, tissue bleaching was required. Bleaching can cause tissue damage [41] and quenching of endogenous fluorescence or fluorescent proteins used to label tissues [42]. However, after bleaching the autofluorescence of tissues remains for visualisation of anatomical structures. The ECi methodology sufficiently cleared all sections of the chicken embryo, except the gall bladder. Insufficient clearing of the gall bladder could be overcome by extending the incubation period in hydrogen peroxide; however, the imperfect clearing of this organ did not inhibit sufficient penetration of the light sheet to enable imaging of the surrounding regions. The heavy pigmentation of the eye can inhibit image acquisition without sufficient bleaching, with a previous study requiring injection of hydrogen peroxide directly into a mouse eye for sufficient removal of the ocular pigments [40]. However, the acquisition of high-resolution images of the eye and proximal tissues in this study demonstrated sufficient clearing and penetration of light for image collection without the requirement of hydrogen peroxide injection.

Histological visualisation of the development of the chicken embryo has previously been described in depth [6]. The analysis was performed on embryos at early embryonic stages, with a focus on the development of anatomy and organ systems within the first two weeks of incubation. Only one embryo was visualised later than 13 doi and achieved comparable results to the current study [6]. Similar imaging of entire, late-stage chicken embryos by LSFM techniques has not been performed, with visualisation methods focussing on embryos prior to 10 doi, or an excised brain from embryos at 16 doi [43, 44]. The larger size of late-stage chicken embryos may require the use of perfusion for tissue optical clearing and sufficient penetration of fluorescent staining which is typically coupled with LSFM imaging [45]. However, their underdeveloped vasculature system may impact the utilisation of this technique.

Histological visualisation achieved in the current study provided microscopic resolution of transverse sections of the torso of chicken embryos, as well as sagittal sections of the head. These sections provided a familiar image of anatomical landmarks that could be used to orientate and identify structures in LSFM images. When compared to the equivalent LSFM sections, visualisation of the head and coelom showed both imaging techniques resolved regions of tissue architecture, although LSFM images were required to provide the 3D structural context of critical anatomical landmarks. Identification of cellular processes such as canalisation and formation of cilia required the cellular resolution of histology in this comparison. The 2D visualisation of each section and throughout the embryo was similar for both H&E histological and LSFM techniques. However, the fluorescence projections can provide greater context to the anatomy as it can be viewed across three axes (XY, YZ, XZ), as opposed to histology’s single sectioned plane.

Light sheet visualisation was also aided by the contrasting levels of autofluorescence of the vasculature throughout the embryo sections. The contrast could be caused by autofluorescence of heme protein from the blood remaining trapped within the vasculature [46]. The lack of this high fluorescence contrast between the vasculature and surrounding tissue is specific to the liver in this study, as the contrast is still visible within the kidneys of the same section. This may be due to the permeable nature of the embryo liver, allowing for blood to more readily diffuse out of the highly vascularised organ during clearing.

Visualisation in 3D provided context to the spatial arrangement and structural architecture of the embryo that the histology sections could not. Volumetric projections produced by the LSFM images furthered structural visualisation by providing clear architecture of the bronchioles in the lung and the turbinates within the nasal cavity, and identification of the pulmonary vein in the lung. Structural relationships between organ systems could be explored with 3D fluorescence projections that is enabled through LSFM.

The thin sections used in histological visualisation can be relatively rapidly fixed and stained to enable 2D analysis at cellular resolution to allow examination of cellular morphology and disease pathophysiology [23]. Examination in 2D lacks the broader systemic architectural context, as demonstrated in this study. Three-dimensional histological images can be achieved through serial sectioning, and subsequent digitalisation and reconstructions of a sample [47–49]. For tissues larger than 5 mm in depth, the number of sections could reach into the hundreds, increasing the opportunity of mechanical trauma and other artefacts during preparation [26]. Comparatively, LSFM can provide structural information in both two and three axes with limited sectioning and limiting the risk of damage to tissues during preparation. Light sheet based immunostaining and TOC techniques can target specific structures or pathogens and track networks throughout entire organisms [35,50]. Sample preparation time for LSFM is more time consuming due to the larger sample volume and lacks the same subcellular resolution of histopathology. Combining histopathology and LSFM can therefore provide a more valuable approach to understanding the relationships between cellular structures and tissue anatomy.

The successful application of TOC and LSFM techniques to the chicken embryo in this study provides a foundation of further exploration of these methods and their utilisation for virological and bacteriological research. The chicken embryo is a widely used model for isolation and propagation of infectious agents and tumours [13,16]. The ability to image entire embryos without the requirement of sectioning will aid in the investigation of the distribution of infectious agents throughout the egg and embryo with the use of fluorescent staining [35]. Although novel in the application of LSFM imaging to the late-stage chicken embryo, the current study uses resolution achieved with autofluorescence alone. The addition of perfusion fixation, delipidisation and decalcification techniques to the protocol described could improve the resolution and remove the requirement of bleaching [35,43,51].

Overall, this study succeeded in the application of a tissue clearing technique for visualisation of late-stage chicken embryos. The acquired LSFM images produced high resolution, 3D fluorescence intensity projections, allowing visualisation of the anatomical architecture and providing a greater understanding of the spatial arrangement of organs and their physiological relationships. When coupled with the cellular resolution attained by histological staining, developmental processes such as canalisation in the nasal passage was able to be conclusively identified. This study has provided a foundation for further research into the chicken embryo and *in ovo* infections, enabling the visualisation of disease pathophysiology using both 2D and 3D techniques.

## Supporting information

S1 Video Rotation animation of LSFM acquired 3D fluorescence projection of a transverse cranial coelom section from day 17 chicken embryo.
Volumetric projections of tissues of the lungs (yellow), left pulmonary vein (dark blue), and heart (red). Reconstruction of tissues performed using non-specific tissue autofluorescence at 561nm. Maximum intensity projections at timestamp (mm:ss) 00:08 show 2D cross sections of the tissue along the XY and YZ planes.
(MP4)

S2 Video Rotation animation of LSFM acquired 3D fluorescence projection of a transverse mid-coelom section from day 17 chicken embryo. Volumetric projections of tissues of the kidneys (magenta) and liver (red). Reconstruction of tissues performed using non-specific tissue autofluorescence at 561nm. Maximum intensity projections at timestamp (mm:ss) 00:10 show z-stack images of the tissue.
(MP4)

S3 Video 360 rotational video of right half of the head from a day 17 chicken embryo. Tissue morphology was reconstructed using non-specific tissue autofluorescence (cyan, 561nm). Cranial beak and nasal passage rendered in cyan at timestamp (mm:ss) 00:09 with cross section of conchae reconstruction shown moving from rostral to caudal.
(MP4)
